# Validation of GLIM criteria on malnutrition in older Chinese inpatients

**DOI:** 10.3389/fnut.2022.969666

**Published:** 2022-09-15

**Authors:** Tong Ji, Yun Li, Pan Liu, Yaxin Zhang, Yu Song, Lina Ma

**Affiliations:** Department of Geriatrics, Xuanwu Hospital, Capital Medical University, National Research Center for Geriatric Medicine, Beijing, China

**Keywords:** malnutrition, frailty, GLIM criteria, nutritional screening tools, hospitalized older patients

## Abstract

**Objective:**

Malnutrition is a nutritional disorder and common syndrome that has a high incidence and is easily ignored in hospitalized older patients. It can lead to multiple poor prognoses, such as frailty. Early identification and correct evaluation of possible malnutrition and frailty are essential to improve clinical outcomes in older patients. Therefore, our objective was to explore the applicability and effectiveness of the Global Leadership Initiative on Malnutrition (GLIM) criteria for identifying malnutrition in older patients.

**Methods:**

In total, 223 participants aged ≥60 years were involved. Nutrition was evaluated using the Mini Nutritional Assessment-Full Form (MNA-FF) and GLIM criteria, which adopt a two-step procedure. The first step was to use three different methods for the screening of nutritional risk: the Nutrition Risk Screening 2002, the Mini Nutritional Assessment Short Form (MNA-SF), and the Malnutrition Universal Screening Tool. The second step was to link a combination of at least one phenotypical criterion and one etiological criterion to diagnose malnutrition. The Clinical Frailty Scale was used to assess frailty. Sensitivity, specificity, Youden index, kappa values, and positive and negative predictive values were used to evaluate the validity of the GLIM criteria. Logistic regression models were used to assess whether there was a correlation between malnutrition, as defined by the GLIM criteria, and frailty.

**Results:**

We found that 32.3–49.8% of our patient sample were at risk of malnutrition based on the GLIM diagnosis and using the three different screening tools; 19.3–27.8% of the patients were malnourished. GLIM criteria with MNA-SF as a diagnostic validation and MNA-FF as a reference showed high consistency (*K* = 0.629; *p* < 0.001), sensitivity (90.5%), and specificity (86.4%). Logistic regression analysis showed that malnutrition, using MNA-SF with the GLIM criteria, was relevant for a higher likelihood of frailty (OR = 1.887; 95% CI 1.184–2.589).

**Conclusions:**

The incidence of GLIM-defined malnutrition was 19.3–27.8% using different screening tools. The consistency between the GLIM criteria using the MNA-SF and the MNA methods was high. Malnutrition, as diagnosed by the GLIM criteria with MNA-SF, was significantly correlated with frailty. GLIM criteria with MNA-SF may be a more reliable malnutrition assessment process in older inpatients.

## Introduction

Various major internal and external risk factors, such as physiological dysfunction, comorbidity, polypharmacy, social isolation, economic factors, and lifestyle, can affect the nutritional status of older patients and make them more vulnerable to malnutrition (1–3). Malnutrition, which frequently occurs in older patients, is associated with frailty, sarcopenia, longer length of stay, and increased mortality (4–6). These adverse outcomes impose a heavy social and economic burden. Despite this, malnutrition in hospital settings cannot be accurately diagnosed and treated over time (7). However, there was a lack of global uniform diagnostic standards for malnutrition until 2019, when the Global Leadership Initiative on Malnutrition (GLIM) criteria were announced (8). There are many difficulties in verifying the reliability and practicality of the GLIM consensus due to the lack of a *gold standard*, which requires substantial verification.

The guideline for the validation of the GLIM performance standard recommends that the Mini Nutritional Assessment Full Form (MNA-FF) can be adopted as a *semi-gold standard*. It is stated that the correlation between malnutrition and frailty using verification of the GLIM criteria in older adults is demonstrable (9). The GLIM process consists of two steps: initial screening for nutritional risk and subsequent evaluation of the diagnosis of malnutrition. Step one uses a validated tool for risk screening. However, it is impossible to use several nutritional screening tools for older adults when they are admitted to hospital. Therefore, it is crucial to find the most effective. Malnutrition and frailty share common mechanisms and the same items in screening tools, such as weight loss and impaired function (10). Various recent reports have shown that the Clinical Frailty Scale (CFS) can diagnose a population of older hospitalized patients with a risk of a poor prognosis, such as prolonged hospital stay, increased dependency and death (11–13). Therefore, we believe the GLIM approach demonstrates a stronger association between malnutrition and frailty and that this method is more appropriate.

With the factors mentioned earlier, our objective was to identify which screening tool can better assess malnutrition as the first step of the GLIM process and to evaluate the correlation between GLIM-defined malnutrition and frailty in older Chinese inpatients.

## Subjects and methods

### Participants

Study data were gathered from the SMART database at Xuanwu Hospital Capital Medical University. SMART is an age-based patient cohort study that contains information from comprehensive evaluations that include nutrition, physical performance, functional status, mental-psychological function, frailty, quality of life. The study's inclusion criteria was age ≥60 years and the participants ability to voluntarily sign an agreement. Participants, recruited from July 2019 to April 2022, were assessed for nutritional status and frailty by trained researchers. Demographic, anthropometric, and hospital data, such as comorbidities, medication, and laboratory indicators, were also collected from patients and the electronic medical record system.

### Nutrition assessment

Different instruments and methods were used to evaluate the nutritional status of the subjects. The MNA-FF was adopted to assess nutritional status, which is composed of 18 questions within four domains, anthropometry, general condition, dietary status, and a self-evaluation (14). Participants were regarded as well-nourished (>23.5 points), at risk of malnutrition (17–23.5 points), or malnourished (<17 points). We used the MNA-SF, a straightforward, six-question screening tool for food intake, unintended weight loss, mobility, medical history, and anthropometric measurements. Participants were identified as malnourished (0–7 points), at risk of malnutrition (8–11 points), and normal nutritional status (12–14 points) (15). Nutrition Risk Screening 2002 (NRS-2002) was adopted to evaluate nutritional risk and malnutrition in participants within 48 h after admission to hospital, as recommended by the guidelines of the European Society for Clinical Nutrition and Metabolism (ESPEN). It involves two categories: severity of the disease and poor nutrition and is adjusted for ≥70 years according to age. The scores for both categories ranged from 0 to 3 points. An NRS-2002 score of ≥3 suggest that the patient is at risk of malnutrition (16). We applied the Malnutrition Universal Screening Tool (MUST) to determine older inpatients at risk of malnutrition (17). It consists of three clinical parameters: body mass index (BMI), weight loss within the last 3 to 6 months, and fasting or insufficient intake for more than 5 days due to an acute disease. Participants may be classified as having a low risk of malnutrition (0 points), moderate risk of malnutrition (1 point), or high risk of malnutrition (≥2 points) on the score criteria. Supplementary Table 1 shows in detail the different methods used for nutritional assessment. Anthropometric measurements included weight, height, mid-arm muscle circumference (MAMC) and calf circumference (CC).

### GLIM criteria

The GLIM criteria is a two-stage assessment tool to diagnose adults who are malnourished (Supplementary Table 1). The important first step in nutritional assessment is the screening for risk of malnutrition to determine the 'at risk' status using any verifiable screening tool. We used three different tools, namely NRS-2002, MNA-SF, and MUST, to conduct the first step in the investigation. The second step was to link at least one phenotypical criterion and one etiological criterion. Phenotypical criteria included unintentional weight loss, low BMI, and decreased muscle mass. Reduced muscle mass is defined as the appendicular skeleton muscle index (ASMI). The specific threshold value for ASMI recommended by the Asian Working Group for Sarcopenia (AWGS) was used. ASMI <7 kg/m^2^ in men and ASMI <5.7 kg/m^2^ in women. The value of ASMI is estimated using the formula verified by the gold standard of the dual light energy X-ray absorption method in the Asian population (18); the specific formula is as follows:


$$\begin{array}{l} \text{ASMI}=0.193\times \text{weight}+0.107\times \text{height}-4.157\times \text{gender}\\ \;-0.037\times \text{age}-2.631 \end{array}$$

gender: 1 for men and 2 for women

Etiological criteria include decreased food intake, impaired absorption, disease burden, and inflammation. For more details, see Table 1. We adopted serum C-reactive protein (CRP) levels >10 mg/L as a biomarker to assess inflammation (19).

**Table 1:** Baseline characteristics of study participants.

**Variables**	**All patients**	**Model B**			***P***
	***n* = 223 (100%)**	**Well-nourished**	**Malnutrition risk only**	**Malnutrition**	
		***n* = 112 (50.2%)**	***n* = 49 (22.0%)**	***n* = 62 (27.8%)**	
Age, years	74.74 ± 10.00	70.84 ± 7.95	75.06 ± 8.97	81.55 ± 10.50	***
Female (%)	104 (46.6%)	47 (45.2%)	25 (24.0%)	32 (30.8%)	n.s.
Height (cm)	164.92 ± 8.31	164.35 ± 8.16	166.08 ± 8.47	165.05 ± 8.48	n.s.
Weight (kg)	66.88 ± 13.06	70.07 ± 11.83	69.81 ± 11.01	58.80 ± 13.37	***
BMI (kg/m^2^)	24.51 ± 4.04	25.8462 ± 3.25	25.31 ± 3.78	21.45 ± 3.94	***
ASMI (kg/m^2^)	6.92 ± 1.22	7.11 ± 1.14	7.33 ± 0.94	6.26 ± 1.31	***
MAMC (cm)	26.69 ± 3.82	28.22 ± 3.00	27.16 ± 3.46	23.54 ± 3.58	***
CC (cm)	33.37 ± 4.52	35.22 ± 3.45	33.53 ± 3.29	29.92 ± 5.08	***
Smoking history, n (%)	84 (37.7%)	44 (52.4%)	18 (21.4%)	22 (26.2%)	n.s.
Drinking history, *n* (%)	68 (30.5%)	36 (52.9%)	15 (22.1%)	17 (25.0%)	n.s.
**CCI category**, ***n*** **(%)**
No comorbidity (0)	33 (14.8%)	21 (63.6%)	8 (24.2)	4 (12.1%)	***
Medium-low (1, 2)	95 (42.6%)	64 (67.4%)	19 (20.0%)	12 (12.6%)	
High (≥3)	95 (42.6%)	27 (28.4%)	22 (23.2%)	46 (48.4%)	
CFS Score
Fit and managing well (1–3)	95 (42.6%)	72 (75.8%)	15 (15.8%)	8 (8.4%)	***
Vulnerable or mild frailty (4, 5)	69 (30.9%)	32 (46.4%)	18 (26.1%)	19 (27.5%)	
Moderate frailty (6)	32 (14.3%)	7 (21.9%)	13 (40.6%)	12 (37.5%)	
Severely to very severely frail (7, 8)	22 (9.9%)	1 (4.5%)	3 (13.6%)	18 (81.8%)	
Terminally ill (9)	5 (2.2%)	0 (0.0%)	0 (0.0%)	5 (100.0%)	

Notes: Model B: MNA-SF to GLIM, patients at risk identified by the MNA-SF. BMI, Body Mass Index; ASMI, Appendicular Skeletal Muscle Index; MAMC, Mid-arm Muscle Circumference; CC, Calf Circumference; CCI, Charlson Comorbidity Index; CFS, Clinical Frailty Scale.

***p < 0.001, **p < 0.01, *p < 0.05. n.s.: non-significant.

We used “model A” to represent “NRS-2002 to GLIM”, “model B” to represent “MNA-SF to GLIM”, and “model C” to represent “MUST to GLIM”.

### Frailty assessment

We used CFS, a nine-point frailty screening tool, and verified it as a predictive factor of hospitalization and death (20) to assess frailty in older patients. They may be classified as fit and managing well (1–3 points), vulnerable or mild frailty (4–5 points), moderate frailty (6 points), serious to very serious frailty, and critically ill (9 points) according to the standard score.

### Statistical analysis

Data were analyzed using SPSS (version 25). Normally distributed data were assessed using a one-way analysis of variance (ANOVA) and mean ± standard deviation to express the results. Non-normally distributed data were assessed using the Kruskal-Wallis test, and the Chi-square test was adopted to classify the variables; the results are shown as numbers and percentages. MNA is a *semi-gold standard* for malnutrition in older adults (9); therefore, sensitivity (SE), specificity (SP), Youden index, kappa values, positive predictive value (PPV) and negative predictive value (NPV) were used for the consistency and effectiveness of the GLIM criteria. The receiver operating characteristic (ROC) curves of NRS-2002, MNA-SF, and MUST were also analyzed to assess their ability to correctly identify malnourished inpatients using MNA as a reference. To evaluate the correlation between nutritional status and frailty, ordered classification logistic regression models were used, the results were expressed as odds ratios (OR) and coefficients with 95% confidence intervals (CI); *P* ≤ 0.05 was taken to indicate statistical significance.

### Sample size calculation

This study was a single-door design diagnostic study. Diagnostic indicators were qualitative: good nutritional status, risk of malnutrition, and malnutrition. PASS was adopted to calculate the sample size. According to previous literature reports, the prior probability is estimated to be 22% (95% CI 18.9–22.5) (21). A systematic review illustrated that the combined sensitivity of the GLIM criteria was 0.72 (95% CI 0.64–0.78), the specificity was 0.82 (95% CI 0.72–0.88) (22), the sample size was estimated using a one-sided test, the class I error was 0.025, and the assurance was 80%. In addition, invalid sensitivity or specificity was set to 0.5. According to the results of the PASS software, 222 samples are needed in this study. At least 42 patients with malnutrition are clearly diagnosed according to the MNA, semi-gold standard, to achieve the statistical efficiency of the detection preset sensitivity. Therefore, the sample size of this study was considered sufficient.

## Results

### Baseline characteristics of the study participants

A sample size of 223 subjects were included in the study. The mean age was 74 years and 46.4% were women. Table 1 illustrates the baseline demographic and clinical characteristics for all patients based on the categorization of model B (Supplementary Table 2 and Supplementary Table 3 show the baseline characteristics reflect the categorization of model A and model C). According to GLIM criteria following the results of the MNA-SF screening, 49.8% of the subjects were at risk of malnutrition and the malnutrition ratio was 27.8%. Significantly, we reported older age, lower body weight, BMI, ASMI, MAMC, CC, and higher CCI scores in the malnourished group than in the group without malnutrition. Regardless of the screening tool used to identify the risk of malnutrition, we found that frailty, defined by CFS, worsened in the malnourished group.

### Agreement between GLIM and MNA

All participants received three nutritional screening tools (NRS-2002, MNA-SF, and MUST) at admission. Cross-tabulation of GLIM criteria after different screening tools and MNA-FF is shown in Supplementary Table 4. The consistency between MNA and model A or model C was regarded as 'moderate' (*k* = 0.493 and 0.526, respectively; *p* < 0.001); however, the consistency between MNA and model B was 'substantial' (*k* = 0.629; *p* < 0.001) (Table 2). SE was 71.4% with model A, 90.5% with model B, and 85.7% with model C, while specificity was 92.5, 86.4, and 90.3% with model A, model B, and model C, respectively. The Youden index for model A was 0.638, while the Youden index was 0.769 for model B and 0.760 for model C (Table 2).

**Table 2:** Estimates of the GLIM models accuracy and precision using the MNA score as reference.

**Diagnosis**	**Model A**	**Model B**	**Model C**
Well-nourished, *n* (%)	151 (67.7%)	112 (50.2%)	140 (62.8%)
Malnutrition risk, *n* (%)	72 (32.3%)	111 (49.8%)	83 (37.2%)
Malnutrition, *n* (%)	43 (19.3%)	62 (27.8%)	53 (23.8%)
SE (%)	71.4%	90.5%	85.7%
SP (%)	92.5%	86.4%	90.3%
Youden index	0.638	0.769	0.760
Kappa (*P*-value)	0.493 (<0.001)	0.629 (<0.001)	0.526 (<0.001)
PPV	69.8%	61.3%	67.9%
NPV	93.3%	97.5%	96.5%
AUC^#^ (95% CI)	0.817 (0.748, 0.886)	0.993 (0.986, 1)	0.860 (0.788,0.931)

SE, Sensitivity; SP, Specificity; PPV, Positive predictive value; NPV, Negative predictive value; AUC, Area Under Curve.

^#^Three screening tools were used to evaluate the ability to accurately distinguish malnourished patients using MNA as a reference.

**Figure 1:**
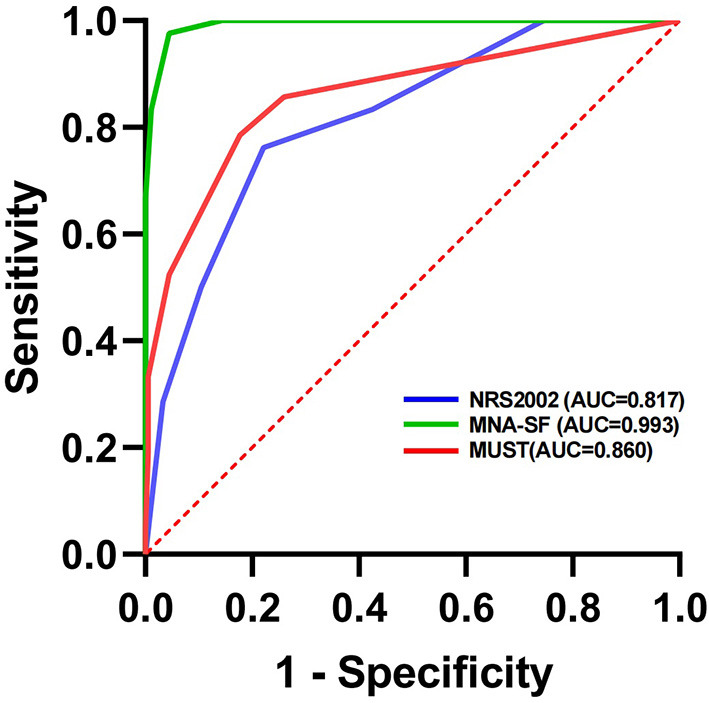
Evaluation of the efficacy of three different screening tools to diagnose malnutrition in aged inpatients using the MNA score as a reference.

The PPVs with model A, model B and model C were 69.8, 61.3, and 67.9%, respectively, while the NPVs were 93.3, 97.5, and 96.5%, respectively. Lastly, the area under the curve (AUC) showed that three different screening tools may have great utility value for diagnosing malnutrition in aged patients (AUC of MUST, MNA-SF, and NRS-2002 were 0.817, 0.993, and 0.860, respectively; Figure 1).

### Malnutrition and frailty

When estimating the relationship between malnutrition and frailty, the CFS used, no matter which method was used to assess nutritional status, a clear association was observed (Table 3). However, there are some subtle differences observed in the results with the intensity of correlation. In all situations (model A, model B, and model C), participants at risk of malnutrition showed an OR of 1.13, 1.55, and 1.24, respectively. For model 1 (model without adjustment) in the scenario of model A, model B, and model C, respectively, offsetting for OR of 0.31, 1.38, and 1.13, and OR of 0.18, 1.31, and 1.01 for the adjusted models 2 and 3 (after adjusting for demographic and clinical parameters), respectively. There was a positive correlation between frailty and malnutrition when nutrition was the only independent parameter (model 1). Whatever covariates were included in models 2, 3, and 4, this significance was observed. Malnutrition, adopted in model B, was associated with a higher risk of frailty incidence, which increased by OR: 2.80 in the case of model 1, by OR: 2.03 in model 2, by OR: 1.89 in model 3, and by OR: 1.91 in model 4 compared to other tools for risk assessment (model A and model C).

**Table 3:** Association between nutritional status (defined by different screening tools to GLIM) and frailty according to the Clinical Frailty Scale.

**Variables**	**CFS**							
	**Model B (Odds Ratio)**				**Model A (Odds Ratio)**				**Model C (Odds Ratio)**			
	**Model 1**	**Model 2**	**Model 3**	**Model 4**	**Model 1**	**Model 2**	**Model 3**	**Model 4**	**Model 1**	**Model 2**	**Model 3**	**Model 4**
**Nutrition status**												
At malnutrition risk	1.552***	1.383***	1.319***	1.306***	1.129***	0.313	0.183***	0.175	1.236***	1.137***	1.013***	1.057**
	(0.915–2.188)	(0.730–2.037)	(0.660–1.977)	(0.647–1.965)	(0.409–1.849)	(–434–1.060)	(–0.571–0.936)	(– 0.571–0.936)	(0.517–1.956)	(0.402–1.873)	(0.267–1.759)	(0.308–1.807)
Malnourished	2.800***	2.030***	1.887***	1.908***	3.032***	1.667***	1.528***	1.514***	2.754***	1.782***	1.678***	1.739***
	(2.146–3.454)	(1.344–2.715)	(1.184–2.589)	(1.204–2.612)	(2.307–3.757)	(0.902–2.432)	(0.750–2.307)	(0.735–2.294)	(2.092–3.416)	(1.089–2.475)	(0.971–2.384)	(1.029–2.450)
Age		0.149***	0.137***	0.140***		0.146***	0.133***	0.135***		0.151***	0.137***	0.139***
		(0.116–0.182)	(0.103–0.172)	(0.105–0.174)		(0.112–0.180)	(0.098–0.168)	(0.100–0.170)		(0.118–0.184)	(0.103–0.172)	(0.105–0.174)
Female		–0.103	0.120	0.120		0.190	0.203	0.186		0.173	0.18	0.172
		(–0.594–0.388)	(–0.374–0.615)	(–0.377–0.616)		(–0.298–0.678)	(–0.289–0.695)	(–0.308–0.680)		(–0.317–0.0.662)	(0.314–0.673)	(–0.324–0.667)
**CCI categories**												
Medium-low (1, 2)			0.393	0.275			0.350	0.209			0.435	0.283
			(–0.365–1.152)	(–0.495–1.046)			(–0.403–1.103)	(–0.557–0.974)			(–0.323–1.194)	(–0.488–1.053)
High (≥3)			1.059*	1.027*			1.175**	1.145**			1.175**	1.093*
			(0.227–1.891)	(0.186–1.868)			(0.348–2.001)	(0.310–1.980)			(0.308–1.967)	(0.254–1.932)
**Lifestyle**												
Smoking history				–0.237				– 0.292				–0.358
				(–0.868–0.393)				(–0.921–0.337)				(–0.989–0.273)
Drinking history				0.717*				0.700*				0.839*
				(0.047–1.386)				(0.033–1.367)				(0.167–0.152)

Model A: NRS-2002 to GLIM, patients at risk identified by the NRS2002. Model B: MNA-SF to GLIM, patients at risk identified by the MNA-SF. Model C: MUST to GLIM, patients at risk identified by the MUST. CFS, Clinical Frailty Scale; CCI, Charlson Comorbidity Index.

95% confidence intervals in parentheses. ***p < 0.001, **p < 0.01, *p < 0.05.

Reference categories: well-nourished, men, no comorbidity.

Model 1 includes being at malnutrition risk or malnourished. Model 2 adds to Model 1 sociodemographic characteristics (age and gender). Model 3 adds to Model 2 the comorbidity according to the CCI. Model 4 adds to Model 3 the lifestyle (smoking and drinking history).

## Discussion

Based on what we know, few studies have adopted MNA-FF as a semi-gold standard to verify the applicability of GLIM criteria and to explore the association between malnutrition and frailty in older patients. To our knowledge, this is the first study to adopt MNA-FF as a reference to verify the reliability of GLIM criteria using different nutrition screening tools for the diagnosis of malnutrition and to analyze its correlation with frailty in older Chinese inpatients. Compared to other screening tools (NRS-2002 and MUST), the most accurate and reliable GLIM diagnostic criteria were obtained using MNA-SF. The prevalence of risk of malnutrition was 49.7% after screening for MNA-SF, while GLIM-defined malnutrition as 27.8%. More importantly, our results showed that nutritional status, defined by GLIM criteria with MNA-SF, was closely correlated with frailty with a higher OR.

We used three different nutritional screening tools to determine the risk of malnutrition, among our participants. NRS-2002 and MUST had similar risk rates of approximately one-third, while nearly half of the patients were at risk as determined by MNA-SF. However, a study from Greece evaluated the predictive value of six different nutritional screening tools for malnutrition in older patients and showed that the malnutrition risk rate with NRS-2002, MNA-SF, and MUST were 97.6, 82.3, and 66.1%, respectively (23). This is much higher than in our study. Possible variances include different regions and different disease severity of the participants. Our study showed that three screening tools could be valid tools to adopt with older inpatients. This result was consistent with a MaNuEL study (24). The incidence of GLIM-defined malnutrition after screening for NRS-2002, MNA-SF, and MUST was 19.3, 27.8, and 23.8%, respectively. The results are consistent with a Chinese study based on hospital malnutrition in older adults by Xu et al. (25), who reported a malnutrition ratio of 27.5% with GLIM using NRS-2002, 32.6% using MNA-SF, and 25.4% using MUST. It could be that this study population was older than 70 years, so the rate will be slightly higher than in our study. We found that the prevalence of malnutrition based on the GLIM process varies with the choice of nutrition screening tools. However, one purpose for the GLIM process is that malnutrition rates can be directly comparable in different studies. To achieve this goal, the GLIM criteria must be further standardized. We need to determine a more suitable screening method for older adults, otherwise it may not be authentic.

Regarding the uniformity between the MNA and GLIM criteria, the MNA-SF seems to have better consistency. In this study, we identified that when MNA-SF was used as a screening tool, we observed the best consistency and applicability between the GLIM criteria and MNA-FF. Our findings are in good agreement with previous studies (25–27). A Chinese study on hospitalized patients over 70 years of age found that when MNA-SF was used as a screening tool, the odds ratio of in-hospital mortality was significantly related to the malnutrition defined by GLIM (25). Munoz et al. showed that GLIM criteria with MNA-SF may be a suitable method for diagnosing malnutrition in older adults in the emergency department (26).

Poor nutritional condition is widely known to be often considered one of the potential mechanisms resulting in frailty in older adults (28). Guidelines on the validation of GLIM criteria recommend that protein-energy malnutrition determined by GLIM criteria related to valid and reliable measures of frailty can be considered an acceptable verification method (9). Our results showed a significant cross-sectional association between frailty and malnutrition, as determined by the GLIM criteria. Similarly, in a study of Spanish older adults living in the community, Rodríguez-Mañas et al. (29) revealed that there was not only a significant horizontal association between GLIM-defined malnutrition and frailty, but also that during the 3-year follow-up period, nutritional condition was a related factor that contributed to the risk of frailty and death. There two studies have also found that malnutrition, based on GLIM criteria, is associated with frailty, and has predictive value among community residents (30, 31). This finding is clinically significant and suggests it can improve nutritional status and prevent or treat frailty. Among the different screening tools, the GLIM criteria adopting MNA-SF as a screening tool may have a better correlation. Contrary to our findings, Yeung et al. (31) found no correlation between malnutrition and frailty in institutionalized older adults. However, Yeung et al. did not choose parameters (e.g., validated body composition measures and inflammatory markers) as part of GLIM criteria. It may not be possible to draw a clearer conclusion about the effectiveness of the predictive validity of the GLIM criteria.

The main strength of this study is the first to adopt MNA-FF as a reference, as recommended by the GLIM validation guide, to verify the reliability of the GLIM criteria using different nutrition screening tools for the diagnosis of malnutrition and to analyze the correlation between malnutrition and frailty in older Chinese inpatients. Moreover, we adopted standardized diagnostic methods for malnutrition based on the GLIM process and rigorous statistical analysis. Our study had limitations. First, it was a single center, small sample retrospective investigation, which can increase bias and lead to restricted subgroup analysis and imprecision. Moreover, we used a formula instead of dual-energy X-ray absorptiometry or bioelectrical impedance analysis to measure muscle mass. Although this formula has been verified, we admit that imprecise muscle mass estimates might fail to guarantee the validity of the results. A further limitation of the study is that we did not compare anthropometric indexes (e.g., ASMI, MAMC, and CC) to verify our results, and we will try to use this method to verify in future research. Furthermore, we did not use a complete nutritional assessment, as a gold standard, to assess the nutritional status of older adults. Because participants with serious diseases in this study may not be able to cooperate with this assessment. We will adopt a complete nutritional assessment of participants in our ongoing study (the 3M study) (32). Finally, this study found a cross-sectional correlation between poor nutritional condition and frailty, and it is impossible to make causal inferences, which must be verified in a larger prospective study.

In this single-center cross-sectional study of older hospitalized patients, the incidence of GLIM-defined malnutrition was 19.3–27.8% using different screening tools. The consistency between the GLIM criteria using the MNA-SF and the MNA methods was high. Malnutrition, as defined by the GLIM criteria with MNA-SF, was significantly correlated with frailty. GLIM criteria with MNA-SF could identify malnutrition more accurately and reliably in older patients. In the future, aim to bring older, malnourished patients, diagnosed by GLIM criteria and with the MNA-SF, into the medical insurance scheme, so that patients can benefit.

## Data availability statement

The original contributions presented in the study are included in the article/supplementary material, further inquiries can be directed to the corresponding author/s.

## Ethics statement

This study was approved by the ethics review board of Xuanwu Hospital Capital Medical University (2021-060). The participants provided their written informed consent to participate in this study.

## Author contributions

TJ drafted and wrote the manuscript. LM and YL designed the study, revised the manuscript and supervised the work. TJ, PL, YZ, and YS gathered the data. All authors contributed to the article and approved the submitted version.

## Funding

This work was supported by the National Key R&D Program of China (2020YFC2008604 and 2020YFC2008600).

## Conflict of interest

The authors declare that the research was conducted in the absence of any commercial or financial relationships that could be construed as a potential conflict of interest. The reviewer QZ declared a shared affiliation with the author to the handling editor at the time of review.

## Publisher's note

All claims expressed in this article are solely those of the authors and do not necessarily represent those of their affiliated organizations, or those of the publisher, the editors and the reviewers. Any product that may be evaluated in this article, or claim that may be made by its manufacturer, is not guaranteed or endorsed by the publisher.

## References

[1] O'Keeffe M Kelly M O'Herlihy E O'Toole PW Kearney PM Timmons S. Potentially modifiable determinants of malnutrition in older adults: a systematic review. Clin Nutr. (2019) 38:2477–98. 10.1016/j.clnu.2018.12.007 30685297

[2] Volkert D Kiesswetter E Cederholm T Donini LM Eglseer D Norman K. Development of a model on determinants of malnutrition in aged persons: a MaNuEL project. Gerontol Geriatr Med. (2019) 5:798990387. 10.1177/2333721419858438 31259204 PMC6589946

[3] Norman K Hass U Pirlich M. Malnutrition in older adults-recent advances and remaining challenges. Nutrients. (2021) 13:2764. 10.3390/nu13082764 34444924 PMC8399049

[4] Lorenzo-Lopez L Maseda A de Labra C Regueiro-Folgueira L Rodriguez-Villamil JL Millan-Calenti JC. Nutritional determinants of frailty in older adults: a systematic review. BMC Geriatr. (2017) 17:108. 10.1186/s12877-017-0496-2 28506216 PMC5433026

[5] Felder S Lechtenboehmer C Bally M Fehr R Deiss M Faessler L. Association of nutritional risk and adverse medical outcomes across different medical inpatient populations. Nutrition. (2015) 31:1385–93. 10.1016/j.nut.2015.06.007 26429660

[6] Khalatbari-Soltani S Marques-Vidal P. The economic cost of hospital malnutrition in Europe; a narrative review. Clin Nutr ESPEN. (2015) 10:e89–94. 10.1016/j.clnesp.2015.04.003 28531387

[7] Cederholm T Barazzoni R A A year with the GLIM diagnosis of malnutrition—does it work for older persons? Curr Opin Clin Nutr Metab Care. (2021) 24:4–9. 10.1097/MCO.0000000000000710 33323713

[8] Jensen GL Cederholm T Correia M Gonzalez MC Fukushima R Higashiguchi T. GLIM criteria for the diagnosis of malnutrition: a consensus report from the global clinical nutrition community. JPEN J Parenter Enteral Nutr. (2019) 43:32–40. 10.1002/jpen.1440 30175461

[9] Keller H. de van der Schueren M Jensen GL Barazzoni R Compher C Correia M. Global leadership initiative on malnutrition (GLIM): Guidance on validation of the operational criteria for the diagnosis of Protein-Energy malnutrition in adults. JPEN J Parenter Enteral Nutr. (2020) 44:992–1003. 10.1002/jpen.1806 32529700

[10] Laur CV McNicholl T Valaitis R Keller HH. Malnutrition or frailty? Overlap and evidence gaps in the diagnosis and treatment of frailty and malnutrition. Appl Physiol Nutr Metab. (2017) 42:449–58. 10.1139/apnm-2016-0652 28322060

[11] Wallis SJ Wall J Biram RWS Romero-Ortuno R. Association of the clinical frailty scale with hospital outcomes. QJM. (2015) 108:943–9. 10.1093/qjmed/hcv066 25778109

[12] Hubbard RE Peel NM Samanta M Gray LC Mitnitski A Rockwood K. Frailty status at admission to hospital predicts multiple adverse outcomes. Age Ageing. (2017) 46:801–6. 10.1093/ageing/afx081 28531254

[13] Basic D Shanley C. Frailty in an older inpatient population. J Aging Health. (2015) 27:670–85. 10.1177/0898264314558202 25414168

[14] Guigoz Y Vellas B Garry PJ. Assessing the nutritional status of the elderly: the mini nutritional assessment as part of the geriatric evaluation. Nutr Rev. (1996) 54:S59–65. 10.1111/j.1753-4887.1996.tb03793.x 8919685

[15] Rubenstein LZ Harker JO Salva A Guigoz Y Vellas B. Screening for undernutrition in geriatric practice: developing the short-form mini-nutritional assessment (MNA-SF). J Gerontol A Biol Sci Med Sci. (2001) 56:M366–72. 10.1093/gerona/56.6.M366 11382797

[16] Kondrup J Allison SP Elia M Vellas B Plauth M. ESPEN guidelines for nutrition screening 2002. Clin Nutr. (2003) 22:415–21. 10.1016/S0261-5614(03)00098-0 12880610

[17] Stratton RJ Hackston A Longmore D Dixon R Price S Stroud M. Malnutrition in hospital outpatients and inpatients: prevalence, concurrent validity and ease of use of the 'malnutrition universal screening tool' ('MUST') for adults. Br J Nutr. (2004) 92:799–808. 10.1079/BJN20041258 15533269

[18] Wen X Wang M Jiang CM Zhang YM. Anthropometric equation for estimation of appendicular skeletal muscle mass in Chinese adults. Asia Pac J Clin Nutr. (2011) 20:551–6. 22094840

[19] Merker M Felder M Gueissaz L Bolliger R Tribolet P Kagi-Braun N. Association of baseline inflammation with effectiveness of nutritional support among patients with Disease-Related malnutrition: A secondary analysis of a randomized clinical trial. JAMA Netw Open. (2020) 3:e200663. 10.1001/jamanetworkopen.2020.0663 32154887 PMC7064875

[20] RittMSchwarzCKronawitterVDelinicABollheimerLCGassmannKG. Analysis of rockwood et al.'s clinical frailty scale and fried et al.'s frailty phenotype as predictors of mortality and other clinical outcomes in older patients who were admitted to a geriatric ward. J Nutr Health Aging. (2015) 19:1043–8. 10.1007/s12603-015-0667-926624218

[21] Cereda E Pedrolli C Klersy C Bonardi C Quarleri L Cappello S. Nutritional status in older persons according to healthcare setting: a systematic review and meta-analysis of prevalence data using MNA((R)). Clin Nutr. (2016) 35:1282–90. 10.1016/j.clnu.2016.03.008 27086194

[22] Huo Z Chong F Yin L Lu Z Liu J Xu H. Accuracy of the GLIM criteria for diagnosing malnutrition: A systematic review and meta-analysis. Clin Nutr. (2022) 41:1208–17. 10.1016/j.clnu.2022.04.005 35504163

[23] Poulia KA Yannakoulia M Karageorgou D Gamaletsou M Panagiotakos DB Sipsas NV. Evaluation of the efficacy of six nutritional screening tools to predict malnutrition in the elderly. Clin Nutr. (2012) 31:378–85. 10.1016/j.clnu.2011.11.017 22182948

[24] Power L Mullally D Gibney ER Clarke M Visser M Volkert D. A review of the validity of malnutrition screening tools used in older adults in community and healthcare settings—a MaNuEL study. Clin Nutr ESPEN. (2018) 24:1–13. 10.1016/j.clnesp.2018.02.005 29576345

[25] XuJYZhuMWZhangHLiLTangPXChenW. A cross-sectional study of glim-defined malnutrition based on new validated calf circumference cut-off values and different screening tools in hospitalised patients over 70 years old. J Nutr Health Aging. (2020) 24:832–8. 10.1007/s12603-020-1386-433009533

[26] Munoz FS Garcez FB Alencar J Cederholm T Aprahamian I Morley JE. Applicability of the GLIM criteria for the diagnosis of malnutrition in older adults in the emergency ward: a pilot validation study. Clin Nutr. (2021) 40:5447–56. 10.1016/j.clnu.2021.09.024 34653825

[27] Donini LM Poggiogalle E Molfino A Rosano A Lenzi A Rossi FF. Mini-Nutritional assessment, malnutrition universal screening tool, and nutrition risk screening tool for the nutritional evaluation of older nursing home residents. J Am Med Dir Assoc. (2016) 17:911–59. 10.1016/j.jamda.2016.06.028 27528452

[28] Hoogendijk EO Afilalo J Ensrud KE Kowal P Onder G Fried LP. Frailty: implications for clinical practice and public health. Lancet. (2019) 394:1365–75. 10.1016/S0140-6736(19)31786-6 31609228

[29] Rodriguez-Manas L Rodriguez-Sanchez B Carnicero JA Rueda R Garcia-Garcia FJ Pereira SL. Impact of nutritional status according to GLIM criteria on the risk of incident frailty and mortality in community-dwelling older adults. Clin Nutr. (2021) 40:1192–8. 10.1016/j.clnu.2020.07.032 32826110

[30] Yeung S Chan R Kwok T Lee J Woo J. Malnutrition according to GLIM criteria and adverse outcomes in community-dwelling Chinese older adults: a prospective analysis. J Am Med Dir Assoc. (2021) 22:1953–9. 10.1016/j.jamda.2020.09.029 33153909

[31] YeungSChanJChanRShamAHoSCWooJ. Predictive value of the GLIM criteria in chinese community-dwelling and institutionalized older adults aged 70 years and over. J Nutr Health Aging. (2021) 25:645–52. 10.1007/s12603-021-1610-x33949632

[32] Ji T Zhang L Han R Peng L Shen S Liu X. Management of malnutrition based on multidisciplinary team decision-making in Chinese older adults (3M study): a prospective, multicenter, randomized, controlled study protocol. Front Nutr. (2022) 9:851590. 10.3389/fnut.2022.851590 35651508 PMC9150743

