# Supplementary material for: Validation of GLIM criteria on malnutrition in older Chinese inpatients
Source: Front Nutr. 2022 Sep 15;9:969666. doi: 10.3389/fnut.2022.969666 (PMC9521176; doi:10.3389/fnut.2022.969666)
Supplement: Supplementary file 1 [file Data_Sheet_1.pdf]

Supplementary Table 1 The different methods for nutrition screening and assessment in the study

• NRS2002

Severity of disease (take the maximum score)

Pelvic fracture, cirrhosis, COPD, long-term hemodialysis, diabetes, and malignant tumor: score 1

Major abdominal surgery, stroke, severe pneumonia, and hematological system tumor: score 2

Craniocerebral injury, myelosuppression, and APACHE＞10 scores: score 3

Nutrition status(take the maximum score)

Good nutrition situation: score 0

Non-volitional weight loss in 3 months＞5% or food intake in the preceding week: < 20-50% of normal requirement: score 1

Non-volitional weight loss in 2 months＞5%, BMI 18.5－20.5 kg/m^2 , or food intake in the preceding week: < 50-75% of normal requirement: score 2

Non-volitional weight loss in 1 months＞5%, BMI ＜18.5 kg/m^2, serum albumin ＜35 g/L or food intake in the preceding week: < 75-100% of normal requirement: score 3

Age ⩾70 years old: score 1

• MNA-SF

A Has food intake declined over the past 3 months due to loss of appetite, digestive problems, chewing or swallowing difficulties?

0 = severe decrease in food intake 1 = moderate decrease in food intake 2 = no decrease in food intake

B Weight loss during the last 3 months

0 = weight loss greater than 3 kg 1 = does not know 2 = weight loss between 1 and 3 kg 3 = no weight loss

C Mobility

0 = bed or chair bound 1 = able to get out of bed / chair but does not go out 2 = goes out

D Has suffered psychological stress or acute disease in the past 3 months? 0 = yes 2 = no

E Neuropsychological problems

0 = severe dementia or depression 1 = mild dementia 2 = no psychological problems

F1 Body Mass Index (BMI) (weight in kg) / $(\text{height in m})^2$

0 = BMI less than 19 1 = BMI 19 to less than 21 2 = BMI 21 to less than 23 3 = BMI 23 or greater

• MUST

BMI: 0=BMI⩾20.0 kg/m^2 1= 18.5⩽BMI＜20.0 kg/m^2 2 = BMI ＜18.5 kg/m^2

Non-volitional weight loss in the last 3-6 months:

0 = within 5% 1=5%-10% 2= More than 10%

Fasting or insufficient intake for more than 5 days due to acute disease:

0=No 2=Yes

• GLIM Criteria

Phenotypic criterion	Nonvolitional weight loss	> 5% within past 6 months or > 10% beyond 6 months
	Low BMI	BMI < 18.5 kg/m^2 for age < 70 years, < 20 kg/m^2 for age ≥ 70 years
	Reduced muscle mass	ASMI < 7.0 kg/m^2 for males, < 5.4 kg/m^2 for females
Etiologic criterion	Reduced food intake	The scores of “0” or “1” on the first MNA-SF item will be considered as positive, or ask about any GI

	symptoms/condition that adversely impacts food assimilation or absorption through the questionnaire.
Inflammatory conditions	Acute disease or injury, or chronic disease-related or CRP > 10 mg/L

COPD: chronic obstructive pulmonary disease; BMI: Body Mass Index; ASMI: Appendicular Skeletal Muscle Index; Gastrointestinal symptoms, such as dysphagia, nausea, vomiting, diarrhea, constipation, or abdominal pain; Chronic conditions, such as short bowel syndrome, pancreatic insufficiency, post-bariatric surgery, esophageal strictures, gastroparesis, or intestinal pseudo-obstruction; Acute disease or injury-related conditions: including major infection, burns, trauma, or closed head injuries; Chronic disease-related conditions: including malignant disease, chronic obstructive pulmonary disease, congestive heart failure, chronic renal disease, or any disease with chronic or recurrent Inflammation.

Supplementary Table 2. Baseline demographic and clinical characteristics of participants

Variables	Model A			P
	Well-nourished n=151 (64.8%)	Malnutrition risk only n=29 (12.4%)	Malnutrition n=43 (18.5%)	
Age, years	71.20 ± 8.68	78.38 ± 7.16	84.74 ± 8.26	***
Female, n(%)	68 (65.4%)	13 (12.5%)	23 (22.1%)	n.s.
Height (cm)	164.8 ± 8.13	164.03 ± 8.36	165.95 ± 8.97	n.s.
Weight (kg)	68.02 ± 12.64	70.12 ± 10.77	60.70 ± 14.24	***
BMI (kg/m^2)	24.95 ± 3.77	26.03 ± 3.24	21.93 ± 4.38	***
ASMI (kg/m^2)	6.96 ± 1.20	7.30 ± 0.98	6.51 ± 1.35	***
MAMC (cm)	27.44 ± 3.55	27.11 ± 3.70	23.76 ± 3.49	***
CC (cm)	34.49 ± 3.94	32.90 ± 3.83	29.76 ± 5.01	***
Smoking history, n(%)	56 (66.7%)	11 (13.1%)	17 (20.2%)	n.s.
Drinking history, n(%)	47 (69.1%)	8 (11.8%)	13 (19.1%)	n.s.
CCI category, n (%)				
No comorbidity (0)	29 (87.9%)	2 (6.1%)	2 (6.1%)	
Medium-low (1－2)	78 (82.1%)	11 (11.6%)	6 (6.3%)	***
High (⩾3)	44 (46.3%)	16 (16.8%)	35 (36.8%)	
CFS Score, n (%)				
Fit and managing well (1-3)	83 (87.4%)	9 (9.5%)	3 (3.2%)	
Vulnerable or mild frailty (4-5)	49 (71%)	9 (13%)	11 (15.9%)	
Moderate frailty (6)	15 (46.9%)	10 (31.3%)	7 (21.9%)	***
Severely to very severely frail (7-8)	4 (18.2%)	1 (4.5%)	17 (77.3%)	
Terminally ill (9)	0 (0.0%)	0 (0.0%)	5 (100%)	

Notes: Model A: NRS2002 to GLIM, patients at risk identified by the NRS2002. BMI, Body Mass Index; ASMI, Appendicular Skeletal Muscle Index; MAMC, Mid-arm Muscle Circumference; CC, Calf Circumference CCI, Charlson Comorbidity Index; CFS, Clinical Frailty Scale.

*** $p<0.001$, ** $p<0.01$, * $p<0.05$. n.s.: non-significant.

Supplementary Table 3. Baseline demographic and clinical characteristics of participants

Variables	Model C			P
	Well-nourished n=140 (62.8%)	Malnutrition risk only n=30 (21.4%)	Malnutrition n=53 (23.8%)	
Age, years	71.66 ± 8.83	74.57 ± 7.00	83.00 ± 9.79	***
Female, n (%)	62 (59.6%)	15 (14.4%)	27 (26.0%)	n.s.
Height (cm)	165.04 ± 8.33	164.53 ± 8.05	164.85 ± 8.54	n.s.
Weight (kg)	69.72 ± 11.94	68.023 ± 11.12	58.73 ± 13.72	***
BMI (kg/m^2)	25.50 ± 3.23	25.22 ± 4.58	21.49 ± 4.22	***
ASMI (kg/m^2)	7.13 ± 1.13	7.10 ± 0.97	6.27 ± 1.36	***
MAMC (cm)	27.91 ± 3.41	26.42 ± 3.16	23.60 ± 3.46	***
CC (cm)	34.69 ± 3.95	33.20 ± 3.90	29.99 ± 4.56	***
Smoking history (%)	54 (64.3%)	10 (11.9%)	20 (23.8%)	n.s.
Drinking history (%)	47 (69.1%)	7 (10.3%)	14 (20.6%)	n.s.
CCI category, n(%)				
No comorbidity (0)	25 (75.8%)	5 (15.2%)	3 (9.1%)	
Medium-low (1 - 2)	76 (80.0%)	9 (9.5%)	10 (10.5)	***
High (⩾3)	39 (41.1%)	16 (16.8%)	40 (42.1%)	
CFS Score ,n (%)				
Fit and managing well (1-3)	81 (85.3%)	8 (8.4%)	6 (6.3%)	
Vulnerable or mild frailty (4-5)	42 (60.9%)	12 (17.4%)	15 (21.7%)	
Moderate frailty (6)	13 (40.6%)	10 (31.3%)	9 (28.1%)	***
Severely to very severely frail (7-8)	4 (18.2%)	0 (0.0%)	18 (81.8%)	
Terminally ill (9)	0 (0.0%)	0 (0.0%)	5 (100.0%)	

Notes: Model C: MUST to GLIM, patients at risk identified by the MUST. BMI, Body Mass Index; ASMI, Appendicular Skeletal Muscle Index; MAMC, Mid-arm Muscle Circumference; CC, Calf Circumference CCI, Charlson Comorbidity Index; CFS, Clinical Frailty Scale.

*** p<0.001, ** p<0.01, * p<0.05. n.s.: non-significant.

Supplementary Table 4 Cross-tabulation of GLIM criteria following different screening tools and MNA-FF.

Tools		MNA-FF			Total
		Well-nourished	Risk	Malnourished	
Model A	Well-nourished	115	26	10	151
	Risk	10	17	2	29
	Malnourished	5	8	30	43
Total		130	51	42	223
Model B		106	6	0	112
		16	29	4	49
		8	16	38	62
Total		130	51	42	223
Model C		112	22	6	140
		14	16	0	30
		4	13	36	53
Total		130	51	42	223

Model A: NRS-2002 to GLIM, patients at risk identified by the NRS2002. Model B: MNA-SF to GLIM, patients at risk identified by the MNA-SF. Model C: MUST to GLIM, patients at risk identified by the MUST.
